# Genetic Diversity and Population Structure of Whitebark Pine (*Pinus albicaulis* Engelm.) in Western North America

**DOI:** 10.1371/journal.pone.0167986

**Published:** 2016-12-16

**Authors:** Jun-Jun Liu, Richard Sniezko, Michael Murray, Ning Wang, Hao Chen, Arezoo Zamany, Rona N. Sturrock, Douglas Savin, Angelia Kegley

**Affiliations:** 1 Pacific Forestry Centre, Canadian Forest Service, Natural Resources Canada, 506 West Burnside Road, Victoria, BC, Canada; 2 USDA Forest Service, Dorena Genetic Resource Center, 34963 Shoreview Road, Cottage Grove, OR, United States of America; 3 Ministry of Forests, Lands and Natural Resource Operations, Nelson, BC, Canada; 4 Qinghai University, Academy of Agriculture and Forestry Science, 253 Ningda Road, Xining, Qinghai, China; USDA Forest Service Rocky Mountain Research Station, UNITED STATES

## Abstract

Whitebark pine (WBP, *Pinus albicaulis* Engelm.) is an endangered conifer species due to heavy mortality from white pine blister rust (WPBR, caused by *Cronartium ribicola*) and mountain pine beetle (*Dendroctonus ponderosae*). Information about genetic diversity and population structure is of fundamental importance for its conservation and restoration. However, current knowledge on the genetic constitution and genomic variation is still limited for WBP. In this study, an integrated genomics approach was applied to characterize seed collections from WBP breeding programs in western North America. RNA-seq analysis was used for *de novo* assembly of the WBP needle transcriptome, which contains 97,447 protein-coding transcripts. Within the transcriptome, single nucleotide polymorphisms (SNPs) were discovered, and more than 22,000 of them were non-synonymous SNPs (ns-SNPs). Following the annotation of genes with ns-SNPs, 216 ns-SNPs within candidate genes with putative functions in disease resistance and plant defense were selected to design SNP arrays for high-throughput genotyping. Among these SNP loci, 71 were highly polymorphic, with sufficient variation to identify a unique genotype for each of the 371 individuals originating from British Columbia (Canada), Oregon and Washington (USA). A clear genetic differentiation was evident among seed families. Analyses of genetic spatial patterns revealed varying degrees of diversity and the existence of several genetic subgroups in the WBP breeding populations. Genetic components were associated with geographic variables and phenotypic rating of WPBR disease severity across landscapes, which may facilitate further identification of WBP genotypes and gene alleles contributing to local adaptation and quantitative resistance to WPBR. The WBP genomic resources developed here provide an invaluable tool for further studies and for exploitation and utilization of the genetic diversity preserved within this endangered conifer and other five-needle pines.

## Introduction

Whitebark pine (WBP, *Pinus albicaulis* Engelm.) is a native keystone conifer species in subalpine ecosystems of western North America. WBP forests provide a food source for animals, reduce soil erosion, and help retain snow in dry and cold mountain regions with steep slopes at high elevations. The ecological roles played by WBP populations are not replaceable by other tree species. Due to threats from white pine blister rust (WPBR) caused by the introduced invasive fungus *Cronartium ribicola* (J.C.Fisch.), mountain pine beetle (*Dendroctonus ponderosae*, Hopkins), altered fire regimes, and climate change [1,2], WBP is designated an endangered species in Canada [3], and has been proposed for listing under the Endangered Species Act in the United States [4].

Loss of WBP populations has been occurring at an increasing rate [5,6]. The continued loss will lead to cascading negative effects on WBP ecosystems, including food loss for wildlife such as Clark's nutcracker (*Nucifraga columbiana*, Wilson), and grizzly bears (*Ursus arctos*, Linnaeus), a decline in biodiversity, and loss of soils and snowpack across subalpine landscapes [1,7–9]. *C*. *ribicola* has now almost spread across the entire distribution of WBP [10], and blister rust infection rates have increased dramatically within much of the range of WBP within the past few decades [11]. As a key component of the WBP genetic restoration program, selection of WBP trees with genetic resistance to WPBR is now underway in the USA and Canada [12, 13]. Major breeding efforts include wild seed collection, seedling inoculation trials to rate WPBR resistance of parent trees, and restoration plantings using WPBR resistant seedlings derived from populations of parent trees with high levels of genetic diversity. There is an urgent requirement to better understand diversity of seed families in selection programs and identify the gene alleles contributing to observed phenotypic variations.

Knowledge about genetic diversity and population structure is important for the restoration of wild WBP populations and the sustainable maintenance of biodiversity and bioprocesses in WBP ecosystems. Efforts to address this need involved characterization of adaptive traits for understanding WBP genetic variation and population differentiation [14–16]. Genetic variation has previously been investigated in WBP collections from different regions using a few types of molecular markers, such as monoterpenes [17], allozymes [18–21], DNAs of mitochondria (mt) and chloroplast (cp) [16,22,23], as well as fragment sequences of orthologous nuclear genes [24]. Decreasing costs for next generation sequencing (NGS) services and advances in high-throughput genotyping technologies have allowed the recent use of targeted capture sequencing to evaluate genetic diversity in WBP stands [25]. All of these studies provide valuable insight into WBP population genetics.

Plant breeding programs usually aim to develop new varieties that have higher productivity and quality, as well as better fitness in habitats undergoing constant changes due to evolving pests/pathogens and shifting climates. In forest tree programs this typically includes maintaining high levels of genetic diversity and adaptation to local environments by capturing a wide range of the adaptive genetic variation of the parents collected for the breeding programs. However, the potential to reach breeding goals depends on the sustainability of the germplasm collected in breeding programs, which is in turn determined by the variability of distinct genotypes and their phylogenetic relationships inside the germplasm. Although efforts on WBP conservation, breeding, and restoration have increased in recent years [12], the WBP genotypes collected so far in western North American regions for the breeding program have not been sufficiently characterized at the molecular level.

This study was undertaken to characterize seed families collected in WBP breeding programs using an integrated genomics approach. Here we report generation of WBP genomic resources by *de novo* assembly of the transcriptome, bioinformatic SNP mining, development of SNP arrays, and application of high-throughput genotyping technology. WBP breeding seed families in western North America were characterized using SNP markers, which may provide a comprehensive insight for efficient management of genetic resources in their ecological restoration. The genomic information and tools will help us understand the underlying patterns of genetic variation in WBP populations across western North America and facilitate WBP genetic improvement for better adaptation to environmental stressors, including enhanced resistance to WPBR and MPB through identification of elite genotypes underlying desirable traits. Maintaining a high level of genetic diversity in WBP restoration populations will help ensure WBP has the potential to continue to evolve in the face of future abiotic and biotic threats.

## Materials and Methods

### Plant materials

For RNA-seq analysis, transcriptome *de novo* assembly, and *in silico* SNP discovery, needles were collected in July, 2013 from one year-old healthy seedlings of 11 open-pollinated seed families growing in a growth chamber at the Pacific Forestry Centre, Victoria, British Columbia (BC), Canada. The seeds for growing these seedlings originated from wild mother cone trees in BC. Needle samples were collected individually for each seedling. Ten seedlings per seed family were pooled when total RNAs were extracted.

**Fig 1:**
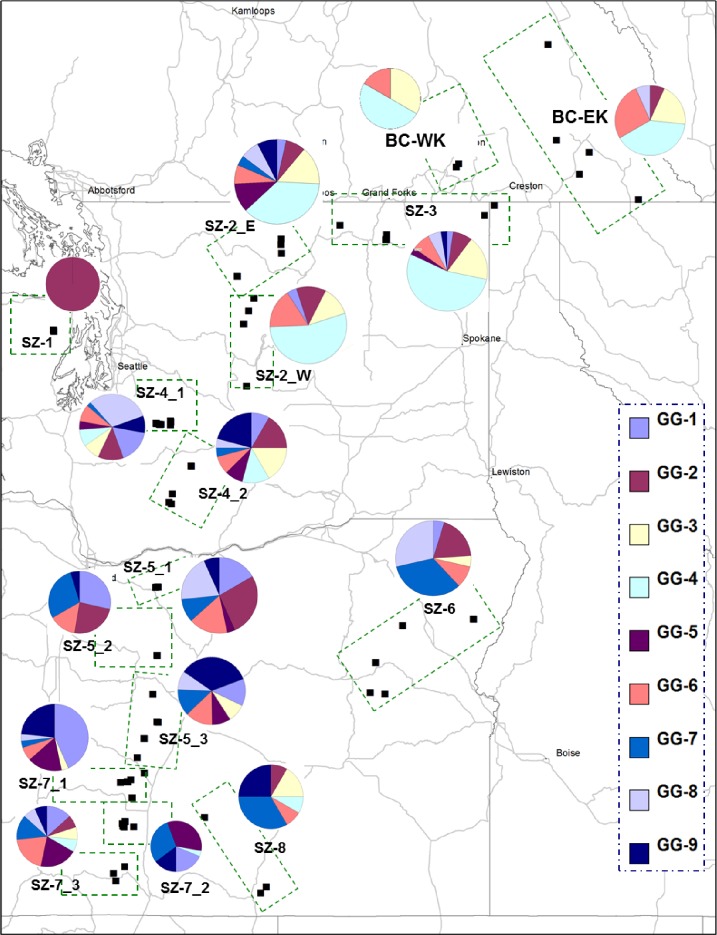
Locations of seed families and geographic distribution of genetic subgroups. A total of 124 seed families were samples in 16 seed (sub) zones. Each pie chart represents the proportion of genetic subgroups (GG-1 to GG-9) as identified by STRUCTURE in a given area.

For verification and application of the SNP genotyping arrays in the population study, we sampled needle tissues for genomic DNA extraction from a total of 372 seedlings from 124 open-pollinated seed families (including seven of 11 BC seed families used for RNA-seq analysis), with three seedlings per seed family. The seeds for these 124 seed families were previously collected from wild mother trees represented in breeding programs. The present work did not include any field studies although WBP is considered as an endangered or protected species. The locations and other related information (geographic coordinates and seed zone assignment) of the seed families are shown in Fig 1 and S1 Table). These seed families originated from two seed planning zones (SPZ) in BC: the West Kootenay (WK) and the East Kootenay (EK); and eight seed zones (SZ-1 to SZ-8) in Washington (WA) and Oregon (OR), USA [2]. For SZ-2, -4, -5, and -7, samples were grouped into two (for SZ-2 and -4) or three (for SZ-5 and -7) subzones because of the large geographical distribution represented in these zones. No specific permissions were required to collect seeds from parent trees for these locations in Canada and USA. This collection covered a total of 16 seed (sub) zones, and represented the wild WBP seed collections that are currently being used in a breeding program to screen for genetic resistance to *C*. *ribicola* at Dorena Genetic Resource Center (DGRC), USDA Forest Service.

Assessment of phenotypic traits relating to quantitative resistance to WPBR was performed at DGRC, with special attention to the number and types of stem infections and overall seedling disease severity [12]. The number of stem infections on individual seedlings varied from 0 to 50 or more. The severity code (0 to 9) denoted the extent of damage from all stem infections (cankers and bark reactions) on the seedling, from none (0) to very extensive (6, 7, 8) to dead from rust (9). This rating was assessed over time as stem infection progressed or was inactivated.

### RNA-seq analysis and *de novo* transcriptome assembly

RNA extractions, cDNA synthesis, and RNA-seq analysis were performed as described previously [26]. Messenger RNA (mRNA) was separated using an RNA-Seq sample preparation kit (Illumina) and used for construction of cDNA libraries with a specific, 6-bp nucleotide bar-coding tags for each sample. Tagged cDNA libraries were pooled in an equal ratio and used for 100 bp paired-end (PE) sequencing on the Illumina HiSeq2500 instrument (Illumina, San Diego, CA, USA) at the National Research Council of Canada (Saskatoon, Canada) in Sept. 2013. The raw Illumina RNA-seq 100-bp PE sequences of WBP needle sample were deposited in the NCBI under BioProject ID: PRJNA352055 with BioSample accession: SAMN05961447, Study accession SRP092411, and SRA run accessions SRR4786281, SRR4786283, and SRR4786284.

Trimmomatic (http://www.usadellab.org/cms/?page=trimmomatic) was used to trim RNA-seq raw reads with default settings at ILLUMINACLIP:TruSeq3-PE.fa:2:30:10 LEADING:3 TRAILING:3 SLIDINGWINDOW:4:15 MINLEN:36 [27]. The trimmed reads from three cDNA libraries were pooled to generate a preliminary WBP needle transcriptome by *de novo* assembly using Trinity, version: trinityrnaseq_r2013-02-25 [28].

The open reading frame (ORF) was predicted by TransDecoder in the Trinity software package at a minimum protein length of 50. To check the WBP proteome predicted from the *de novo* assembled transcriptome, reciprocal BLAST analysis was performed using protein datasets from *P*. *taeda* [29] and *P*. *monticola* [26,30]. Gene annotation was performed using B2G program [31].

### *In silico* SNP detection

To find *in silico* DNA variations in the WBP transcriptome, the CLC Genomics Workbench (v5.5, Arhaus, Denmark) was used to align RNA-seq reads back to the *de novo* assembled transcriptome with parameters: masking mode = no masking; mismatch cost = 2; insertion cost = 3; deletion cost = 3; length fraction = 0.95; similarity fraction = 0.95; auto-detect paired distances = yes; global alignment = yes; non-specific match handling = ignore.

DNA variations (SNP, MNV, and InDel) were then called with parameters: minimum coverage = 10; maximum expected variants = 2; ignore quality scores = no; ignore non-specific matches = yes; ignore broken pairs = yes; variant probability = 90.0; require presence in both forward and reverse reads = yes.

### Selection of ns-SNPs for design of genotyping arrays

Highly differentiated genetic variants are more informative per locus than randomly chosen markers. To design genotyping arrays, SNPs within the WBP transcriptome were evaluated as previously reported [26,32]. We selected SNPs based on their variant types (non-coding *vs*. coding regions, synonymous *vs*. non-synonymous), gene groups where they were localized, and gene expression patterns. The ns-SNPs were selected from candidate groups with putative gene functions in plant disease resistance, defence, or adaptation. Candidate gene groups were determined based on above GO analysis and BLAST analysis against local databases of the *P*. *taeda* proteome derived from a genome sequence draft [29], *P*. *monticola* resistant gene analogs (RGA) of the NBS-LRR and RLK gene families [26], as well as *P*. *monticola* defence-related genes in response to *C*. *ribicola* infection [30]. The ns-SNPs that resulted in dramatic changes in the biochemical properties of amino acids (for example, changes between neutral and acidic or basic amino acids) were considered as candidates of “functional SNPs” and included in genotyping arrays.

A total of 216 ns-SNPs, each per unigene with putative function, were selected for design of Sequenom iPLEX arrays [33]. Multiplex SNP assays were designed using the MASSARRAY® Assay Design software (Sequenom, San Diego, CA, USA) with default parameters. Genomic DNA was extracted and purified from needle tissues individually using a QIAGEN DNeasy plant mini kit (Qiagene, CA, USA). SNP genotyping was performed as previously described [26,32] using a Sequenom iPlex MASSARRAY platform at Laval University (Quebec City, Canada).

### Genetic diversity and cluster analysis

GenAlex v6.5 [34] was used to calculate sample sizes (N), number of alleles (Na), number of effective alleles (Ne), Shannon’s information index (I), expected, observed, or unbiased expected heterozygosity (Ho, He, and uHe), fixation index (F), percentage of polymorphic loci (P), the inbreeding coefficient within individuals relative to the total (Fit), and genetic differentiation among populations (Fst).

At the population level, the genetic differentiation index (Fst) was estimated with a confidence interval of 95% for 999 permutations. The pattern of allelic differentiation among populations was explored through principal coordinate analysis (PCoA) based on the genetic distance matrix with data standardization using GenAlex v6.5. The software POPTREE2 [35] was used to construct phylogenetic trees using Nei’s standard genetic distance among seed (sub) zones with sample size correction (Dst) [36]. DARwin 6.0.12 statistical software [37] was used to determine the genetic dissimilarity among all genotyped individual trees based on Jaccard’s coefficient to draw an unweighted neighbour joining tree.

### Population structure analysis

The Bayesian approach was used to infer the genotype structure without introducing any a priori classification using the program STRUCTURE v2.3.4 [38]. The admixture model was used for the SNP co-dominant loci with 5,000 burn-in length and 50,000 Markov chain Monte Carlo (MCMC) replicates. Twenty simulation runs were performed with K values set from 1 to 30 to estimate the cluster number (K). The most likely number of clusters was then determined using the Delta-K method [39]. The coefficient of membership (Q-matrix) of each individual was assessed with regard to the inferred genetic subgroup.

## Results

### *De novo* assembled WBP needle transcriptome

Using the Illumina Hiseq-2500 platform, three RNA-seq runs generated a total of 160 million 2 x 100-bp PE reads. After trimming, 129,522 transcripts were *de novo* assembled with N50 of 1,692-bp and an average length of 895-bp using Trinity, and estimated to be expressed from 80,323 unigene sequences in a total length of 55-Mb. Following *de novo* assembly, 97,447 coding DNA sequences (CDS) were detected using TransDecoder, and 53.7% of them were predicated as complete for the open reading frames (ORFs). Other characteristics of the assembled transcriptome were shown in S2 Table. This Transcriptome Shotgun Assembly project has been deposited at DDBJ/EMBL/GenBank under the accession GFBO00000000. The version described in this paper is the first version (129,522 transcripts), GFBO01000000.

A BLAST search against local sequence data sets revealed 58,360 CDS (~60% of total) had significant homologous hits (BLASTp E value < e-5) with the putative *P*. *taeda* proteome [29]. Among them, 24,028 WBP CDS were highly conserved with identical hits (BLASTp E values < e-99) to *P*. *taeda* sequences. When the putative *P*. *taeda* proteome was used as a query to search the WBP transcriptome, 94% of the putative *P*. *taeda* proteome was found to have significantly homologous hits in the WBP transcriptome (S3 Table). These results suggest a relatively high coverage of the WBP transcriptome *de novo* assembled without reference. Using BLAST searches against *P*. *monticola* data sets [26,30], 6,881 and 1,670 WBP transcripts were homologous to the defense-responsive genes and resistance gene analogs (RGAs) respectively.

### SNP distribution in the needle transcriptome

**Fig 2:**
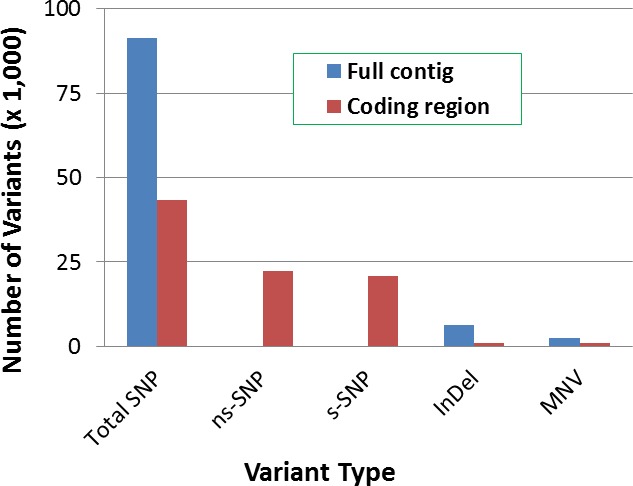
Classification of DNA variation in the whitebark pine transcriptome. Single nucleotide polymorphism (SNP), multiple nucleotide variation (MNV), and small insertions or deletions (InDel) were detected using CLC Genomics Workbench (v5.5). Synonymous and nonsynonymous SNPs (s-SNP and ns-SNP) were determined in the coding DNA sequences (CDS).

*In silico* SNPs were predicted within the WBP needle transcriptome. A total of 100,320 DNA variant sites were identified, 91% of them were SNPs (Fig 2). CDS regions contained 43,248 SNPs, and over half of them (22,291) were non-synonymous SNPs (ns-SNPs), causing amino acid changes or nonsense mutations in the putative ORFs. These ns-SNPs were distributed in 9,529 CDS, which were transcribed from 8,085 unigene sequences.

GO analysis showed that 7,746 ns-SNP containing transcripts had homologous hits in the B2G analysis, and 6,768 of them had at least one GO term. In terms of biological process, these polymorphic genes with putatively functional variations (ns-SNPs) were involved in metabolic processes (2,932), cellular processes (2,556), single-organism processes (1,908), biological regulation (656), response to stimuli (638), regulation of biological processes (569), localization (534), and cellular component organization or biogenesis (387) (S1 Fig).

### SNP genotyping by Sequenom iPLEX technology

Based on the above GO analysis and BLAST search against *P*. *monticola* defense genes in response to *C*. *ribicola* infection, 216 *in silico* ns-SNPs (one per unigene) were selected to design Sequenom iPLEX high-throughput SNP genotyping arrays. After initially screening six panels of SNP arrays (36 SNPs per panel) on a set of 96 samples, 117 ns-SNP loci (54% of total arrays) passed quality control and their genotypes scored successfully across the sample set. Forty-three SNP loci were revealed as homozygous in the set of genotyped samples and three showed low levels of minor allele frequency (MAF ≤ 5%). The remaining 71 ns-SNP loci (S4 and S5 Tables) were considered as informative (MAF > 5%) and selected for genotyping all seedlings sampled in the present study.

### Assessment of genetic diversity using genotypes of ns-SNP markers

The selected 71 ns-SNP markers showed a MAF > 5% across all genotyped samples. Based on their genotypes, genetic diversity was evaluated in the WBP breeding seed collections, which including a total of 371 seedlings of 124 seed families originating from 16 seed (sub) zones in three regions (BC, WA, and OR) (Fig 1). Only one genotyped seedling was excluded for further analysis due to missing data for most SNP loci. We analyzed SNP genotypic data based on seed (sub) zone for precise estimation of the level of genetic diversity. S6 Table shows sample size (N), allele no. (Na), effective allele no. (Ne), Shannon’s information index (I), observed, expected, and unbiased heterozygosity (Ho, He, and uHe), fixation (F), and percentage of polymorphic loci (P).

The comparison of mean expected heterozygosity (He) of alleles across 16 (sub) zones revealed that BC-EK seed families were highly heterozygous (0.40 ± 0.02). In contrast, heterozygosity of the seed families inside SZ-1 (0.26 ± 0.02) was scored at the lowest level (S6 Table). Correspondingly, percentage of polymorphic loci (P) varied from 73.61% (SZ-1) to 97.22% (SZ-2_E, SZ-3, SZ-4_2, and SZ-5_1) with mean of 93.06 (±1.48) %. Genotyped SNP loci were highly polymorphic with sufficient variation to enable unique identification of each individual in all populations, and no identical genotype from 71 ns-SNPs was shared by any two individuals, even those of the same seed family. All of these measurements indicated a high genetic diversity inside each seed (sub) zones.

As a measurement of excess homozygosity, fixation index (F) for all trees within a (sub) zone varied from -0.06 (± 0.05) (SZ-1) to 0.28 (± 0.03) (SZ-4_1) with a mean value of 0.09 (± 0.01), suggesting a general pattern of random mating. In a consistent trend, low mean values for Fis (0.102 ± 0.025), Fit (0.180 ± 0.024), Fst (0.088 ± 0.004), and a relatively high value for Nm (3.092 ± 0.192) were calculated based on all genotyped SNP loci (S7 Table). These results indicate that inbreeding was limited due to out-crossing with high level of gene-flow, but there may be significant differentiation among WBP populations based on Nm and Fst values.

### Phylogenetic relationships among populations

**Fig 3:**
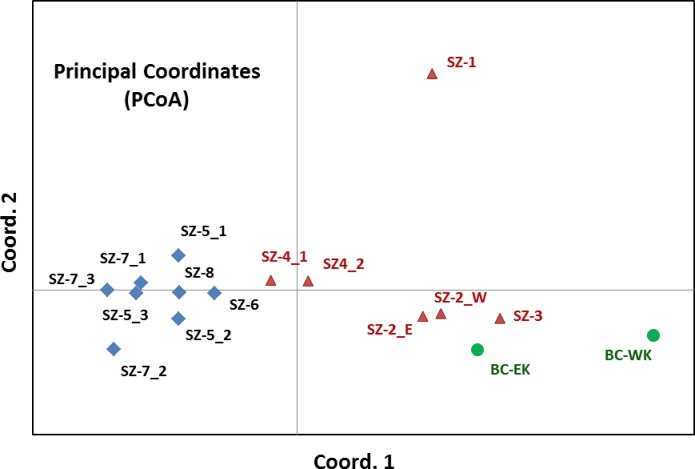
Principal coordinate analysis (PCoA) of whitebark pine populations using GenAlEx version 6.5. Seed zone (subzone) designations were listed in S1 Table. Three regions are shown by colors, Green: British Columbia (BC), Canada; red: Washington State (WA); blue, Oregon state (OR), USA.

Pair-wise genetic distances were measured and used for two-dimensional (2-D) PCoA using GenAlEx (Fig 3). PCoA plot grouped the 16 seed (sub) zones based on their corresponding geographical locations in three regions (BC, WA, and OR). The first principal coordinate (Coord. 1) accounted for 22.77% of total variation, separating seed families into two groups based on their latitude distribution: one with OR and southern WA seed zones (SZ-4 to SZ-8) and the other with three seed zones (SZ-1 to SZ-3) in northern WA and seed families in BC. The second principal coordinate (Coord. 2) accounted for 20.72% of the total variation, clearly separating SZ-1 (seed families at the Olympic National Forest in WA) from all others.

**Fig 4:**
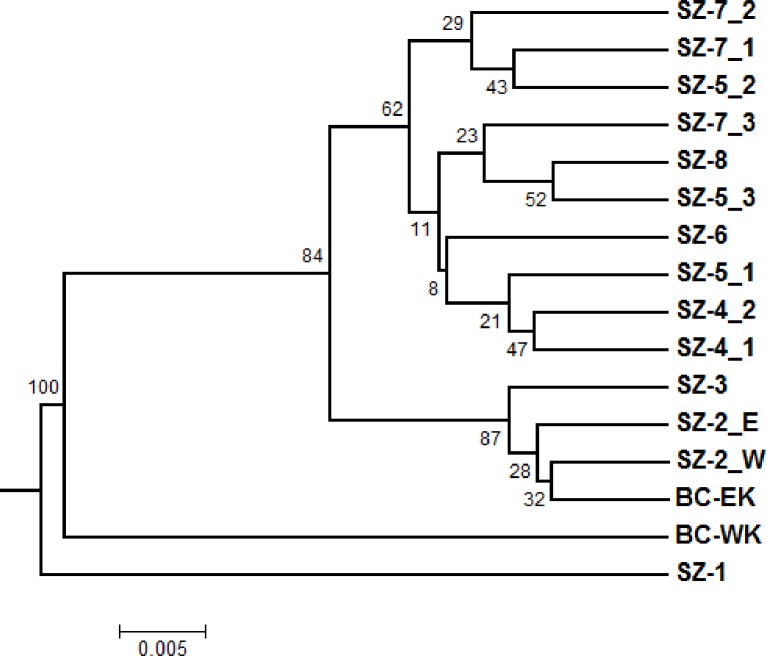
Phylogenetic relationships among whitebark pine populations. Nei’s standard genetic distances with sample size correction (Dst) (Nei 1972) were calculated using genotypic data of 71 SNP loci. A consensus dendrogram was constructed using the unweighted pair-group method with arithmetic mean (UPGMA). Bootstrap values are indicated on the nodes as percentages as tested with 1000 bootstrap replicates.

This 2-D clustering pattern was further supported by a phylogenetic analysis using matrix of Nei’s standard genetic distances with sample size correction (Dst) (Fig 4). A phylogenetic tree, constructed by UPGMA clustering, tended to group geographically local seed families into two major clusters, which were well supported by bootstrap test (62% to 100%). In the UPGMA-based consensus dendrogram, one major cluster grouped four seed zones in southern WA and OR regions together. Although this cluster was divided into three phylogenetic sub-groups, the low bootstrap support among sub-clusters suggests limited resolution for differentiation among those seed (sub) zones in southern WA and OR regions. In another major cluster, BC-EK seed families were grouped with SZ-2 and SZ-3 samples. In contrast, seed families from BC-WK and SZ-1 stood alone as monophyletic groups.

### Analysis of molecular variance (AMOVA) and population structure

**Fig 5:**
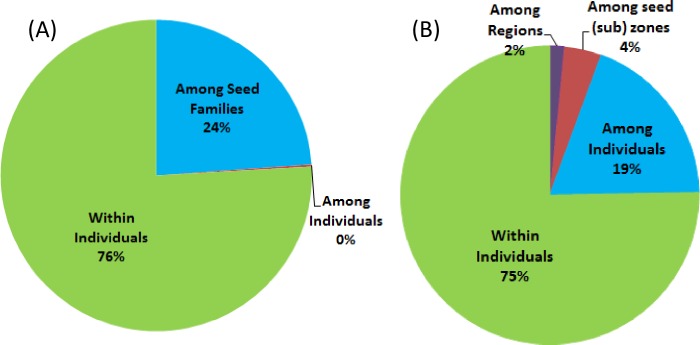
Analysis of Molecular Variance (AMOVA) of whitebark pine samples collected in western North America for a breeding program. (A) AMOVA based on seed families. (B) AMOVA based on seed (sub) zones.

AMOVA was further used to evaluate genetic differentiation (Fig 5). Of the total genetic variation, 24% were detected among seed families and 76% were detected within individuals, but almost no variation was detected among individuals of the same seed family (Fig 5A). When seed (sub) zones and regions (BC, WA, and OR) were considered, the variation among seed families was sub-partitioned. Differentiation among the seed (sub) zones was significant and explained 4% of total variation; 2% of total variation was detected among three regions (BC, WA, and OR). The remaining variation (~19%) resided among individuals (Fig 5B), indicating an important component of genetic variation for wind pollinated and highly outcrossing taxa such as pines.

**Fig 6:**
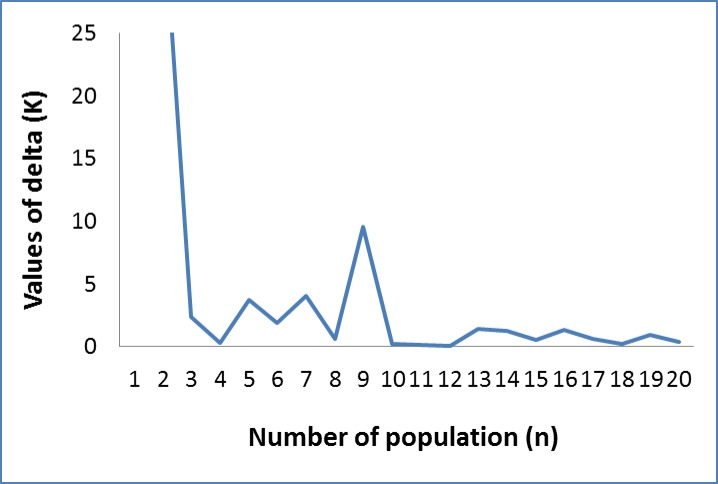
Bayesian clustering analysis for estimation of population structure using program STRUCTURE. ΔK plots by Evanno’s method. Graph of delta K values (y-axe) against assumed sub-populations (x-axe) showing the ideal number of groups present in a set of whitebark pine seed families collected for a breeding program. Genotypic data were collected for 71 ns-SNP loci across all genotyped individuals. The highest peak shows the best K = 9.

The possible WBP population structure was explored among all genotyped samples without introducing any a priori classification using the Bayesian clustering approach implemented in the program STRUCTURE. An admixture model was used here because of the potential that individuals may have mixed ancestry and the presence of gene-flow between different geographical areas. This genotype-based classification provided data for a biological interpretation of the sub-population structure in addition to the geographical origins and classification of the seed zones. There was clear evidence of sub-structuring within all genotyped individual seedlings. The highest peak of ΔK was detected at K = 2 by the Bayesian clustering (Fig 6), supporting the clustering patterns of seed families as revealed by PCoA analysis and UPGMA tree (Fig 3 and Fig 4). Furthermore, the second highest peak of ΔK (at K = 9) was observed (Fig 6), suggesting a population structure comprised of nine genetic subgroups (designated as GG-1 to GG-9) in the collected seed families. This genetic structure of nine subpopulations revealed by STRUCTURE was further supported by analysis of genetic dissimilarity among all genotyped individual trees. Based on Jaccard’s pair-wise dissimilarity coefficient values calculated for SNP data, an unweighted neighbor-joining dendrogram showed a complex clustering pattern (S2 Fig). Therefore, a more detailed analysis was focused at K = 9.

**Fig 7:**
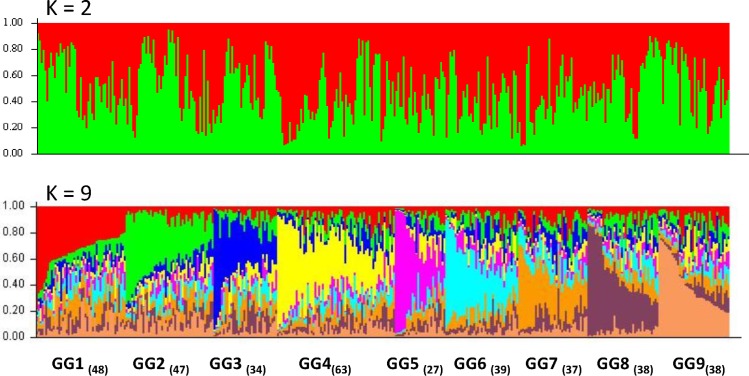
Population structure as shown by bar plot of the estimated membership coefficient (Q) of genotyped samples with SNPs matrix. All genotyped samples were clustered into nine subpopulations (genetic subgroups GG-1 to GG-9). Numbers of individuals assigned to each genetic group are shown in round brackets. Each vertical bar represents one sample, and a subpopulation (genetic subgroup) is shown in one main color. In the vertical bar, the length of each K colored segment corresponds to the proportion of alleles contributed by each of the K subpopulations.

Visual inspection of the STRUCTURE barplots indicated that plots for K = 9 were informative with respect to population substructure (Fig 7). Obviously, each of the nine optimal genetic subgroups has a considerable portion of mixed memberships among groups. Since the Bayesian approach is a quantitative clustering method, we calculated the proportion of the genome of an individual originating from each inferred genetic subgroup. Membership coefficients (the individual Q-matrix) were assessed in each genetic subgroup. The individuals with membership coefficients ≥ 0.5 accounted for 13% (in GG-4) ~ 37% of the total (in GG-5), indicating that a large number of individual trees had a high degree of genetic contributions from multiple genetic subgroups as admixtures.

### Association between genetic diversity and eco-geographical factors

We detected significant relationships between principal components (PC) of genetic variations among seed families and eco-geographical parameters at the site of origin. PC-1 and PC-2 explained 7.82% and 5.50% of the variation at the seed family level, respectively. The most significant correlation was found between PC-1 and latitude (R^2^ = 0.4742, p < 1e-5), followed by the correlation between PC-2 and longitude (R^2^ = 0.0476, p < 0.05) (S3 Fig).

Structure inferred genetic subgroups at K = 9 were assigned to each seed (sub) zone. The spatial distribution pattern of the genetic subgroups across the landscape of three regions (BC, WA, and OR) in western North America is shown in Fig 1, which mimics the patterns as demonstrated in the phylogenetic analysis at the seed (sub) zone level (Fig 3 and Fig 4). Presence of nine genetic subgroups, as revealed by STRUCTURE, indicated that WBP stands of multiple genetic subgroups grow in each seed zone except SZ-1. All genotyped trees in SZ-1 were assigned to GG-2, suggesting that the population in the Olympic National Forest may be relatively isolated from its surrounding areas. In contrast, SZ-2_E, SZ-4, and SZ-7_3 displayed the most complex genetic compositions, comprising all nine genetic subgroups. Compared to OR and southern WA regions, genetic compositions were generally simpler in northern WA and BC regions, where 37% ~ 54% of trees had genotypes belonging to GG-4 (Fig 1).

**Fig 8:**
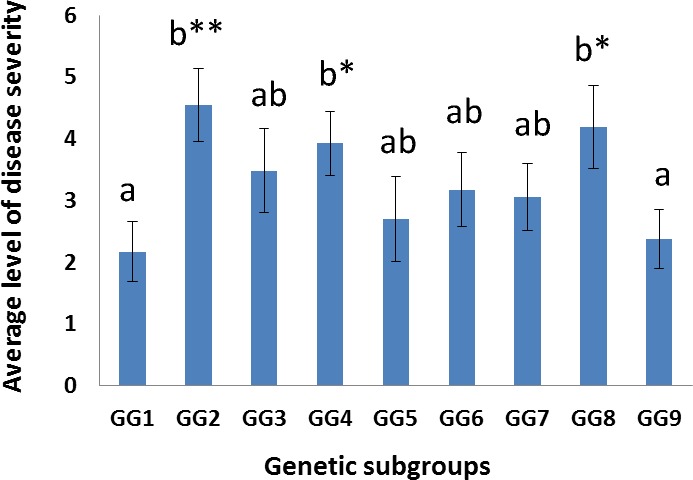
Association of whitebark pine genetic subgroups with relative levels of disease severity post inoculation by *Cronartium ribicola*. (a) Mean values of relative levels of disease severity were shown for seedlings with nine genetic subgroups (GG1 to GG9). Standard error (SE) was calculated based on the entire subpopulation of each genetic subgroup. Statistical difference is significant (T-test and One-way ANOVA test, * *P* < 0.05, ** *P* <0.01) between subgroups labelled with different letters.

Following *C*. *ribicola* inoculation, relative levels of WPBR disease severity were assessed and compared among genetic subgroups. The mean rust disease severity levels of seedling groups with each of nine genetic subgroups (GG1 to GG9) are shown in Fig 7. Individuals of GG2, GG4, and GG8 had the highest mean level of relative disease severity. In contrast, individuals of GG1 and GG9 showed the lowest disease severity levels, significantly lower than those of three groups: GG2, GG4, and GG8 (t-test *P* < 0.05, or *P* < 0.01). The other four subgroups (GG3, GG5-GG7) exhibited medium disease severity levels without significant differences from the others (Fig 8).

## Discussion

### Development of WBP genomic resources by an integrated approach

The development of modern genomic resources can provide baseline knowledge for breeding, conservation, and restoration of endangered organisms. Recent advances in technologies for NGS and automatic SNP high-throughput genotyping have accelerated genomic studies for the characterization of molecular variations in a few five-needle pines. Recently, other investigators identified a large set of *in silico* SNPs in 47 WBP trees by captured targeted sequencing [25]. In addition, restriction-site associated DNA sequencing (RADseq) was used to construct genetic maps of foxtail pine (*P*. *balfouriana* Grev. & Balf.) [40]. RNA-seq analysis using Illumia HiSeq platforms has been applied to the development of genomic resources and molecular tools for breeding programs of western white pine and limber pine [26,30,32,41]. However, limitations are still present for application of cutting-edge technologies to five-needle pine species due to the huge size (20~30 GB) of their highly repetitive genomes [25,29,42].

The present study used an integrated genomics approach to develop a WBP transcriptome and SNP resources. *De novo* assembly of RNA-seq reads generated a WBP needle transcriptome with > 80,000 expressed unigene sequences (S2 Table). The subsequent bioinformatics mining detected about 100,000 SNPs by profiling transcriptomes among seed families from different geographical areas (Fig 2), demonstrating the complexity of the WBP genome. We focused on a subset of informative ns-SNP markers, which revealed phylogenetic relationships and genetic structure among WBP populations in western North America. The genomic variation reported here demonstrates that transcriptome profiling by RNA-seq analysis was an effective strategy for *in silico* SNP discovery throughout a complex genome in a non-model species of conifers. The WBP polymorphic transcriptome may provide invaluable candidates for better understanding of genome-wide gene variations contributing to adaptive traits in future association and functional studies of genes in WBP and other related five-needle pines.

### Application of ns-SNPs in WBP population genetic study

Marker type and sampling size are two important factors in population genetics and molecular breeding. A few types of molecular markers were previously used in WBP population studies with sample collections from various regions [16–24], revealing information on genetic variation levels and geographic or biogeographic patterns at different scales. Due to the limitation of these traditional molecular markers in population investigations, the use of SNP markers has recently become a favourite choice due to their co-dominance, high abundance throughout the whole genome or transcriptome, and suitability to high-throughput genotyping using large populations. Another advantage of SNPs over other molecular markers is that they require much smaller sample sizes. By SNP genotyping, patterns of variability in a population may be reliably captured using as few as four individuals [43]. These features make SNPs ideal for assessing genetic diversity and elucidating phylogenetic relationships and ancestry membership proportions among populations and distribution regions of an organism.

The present study further documented ns-SNPs within candidate genes after evaluating DNA variations (InDel, MNV, and SNP) inside the WBP needle transcriptome. Due to changes in primary protein sequence, ns-SNPs may be more informative per site than SNPs picked up randomly. Several studies revealed that ns-SNPs are more likely to be related to specific biological functions and phenotypes [44,45]. Amino acids are commonly classified into three structural groups based on their side chain at neutral pH: nonpolar, polar but uncharged, and charged (negatively, or positively). Ns-SNPs resulting in amino acid changes between structural groups are presumed as “functional ns-SNPs” (S4 Table).

The candidate gene approach is suited for population genetic studies to detect genes underlying complex traits, i.e. traits for which single candidate genes make a small contribution. A number of candidate genes have been identified as potential targets for selection of adaptive traits in conifers [46,47]. A set of candidate genes cumulatively accounted for ~30% of the phenotypic variance in Sitka spruce cold hardiness and bud set [48]. Multiple pathogenesis-related genes were found in association with quantitative traits of *P*. *monticola* resistance to *C*. *ribicola* in the WPBR pathosystem [49,50].

With these considerations, this study documented over 22,291ns-SNPs (~22% of total SNPs) throughout the WBP transcriptome and annotated genes containing ns-SNPs (Fig 2, S1 Fig). A subset of presumed functional ns-SNPs was genotyped to understand variations of candidate genes with putative involvements in plant defence and adaptation (S4 Table). Over 50% of ns-SNPs were successfully genotyped by Sequenom technology, demonstrating their usefulness in WBP population genetics. With availability of the WBP ns-SNP resource, there is potential for a future study to reveal unique genotypes contributing to WPBR-resistance or other adaptive traits of ecological interest.

### Genetic diversity and population structure

Information about the genetic diversity and population structure in the seed families collected for breeding programs is of fundamental importance for WBP improvement and subsequent restoration efforts. We evaluated genetic diversity using ns-SNPs of 71 candidate genes within and among 124 seed families, which were selected as representative of the WBP breeding materials in regions of BC, WA, and OR. As estimated by expected heterozygosity (He) and percentage of polymorphic SNP loci (P), genetic diversity levels were similar across seed (sub) zones in western North America (S6 Table). The mean He (0.35) was higher in western North American than in the Inland West (He = 0.27) where 147 samples were genotyped using 16 isozymatic loci [16]. Difference of diversity measured in case studies may be caused by different marker types, sampling sizes, and locations [25]. It awaits a more detailed study to determine whether genetic variation is truly at a higher level in western regions than the Inland West due to adaptation to different local habitats.

A mean number of migrants (Nm = 3.092±0.192) calculated in our study was much lower than previous report on WBP populations in the Inland West (Nm = 9.354) [16]. Most conifers have shown to have a large range of numbers of migrants (Nm = 5 ~ 20) [51,52]. A relatively lower Nm value suggests a restricted gene flow, which may be due to geographical isolation and result in subpopulation structuring. Consistent with this speculation, our work found genetic structuring to be at a moderate level (Fst = 0.088) in regions of BC, WA, and OR (S7 Table), higher than previous reports measured by isozymatic loci (Fst = 0.026~0.034) [16,18].

Based on genetic distance, PCoA and UPGMA clustering clearly separated WBP populations from sampled regions into two major groups. One included northern WA and BC, and the other included southern WA and OR (Fig 3 and Fig 4), suggesting that they may have historically originated from different glacial refugia in the south and north Cascades, and Rocky Mountain Range [21,22,23]. We evaluated potential differences in spatial WBP genetic structure at fine and large scales in western North America. ANOVA showed that 24%, 4%, and 2% of the total variances were among seed families, seed (sub) zones, and regions (BC, WA, and OR), respectively (Fig 5). Bayesian clustering consistently detected two main groups and a further nine genetically distinct subgroups (GG-1 ~ GG-9) using the program STRUCTURE (Fig 7). These findings demonstrate an obvious genetic structure in WBP populations, similar to previous reports detailing large geographic scales [14,18,19].

The presence of nine genetically distinct subgroups allowed us to estimate the composition of genetic subgroups in populations. Co-occurrence of all nine genetic subgroups in SZ-2_E, SZ-4, and SZ-7_3 (Fig 1) suggests that the southern-most, middle, and northern-most regions of the Cascades may be migrant fusion zones as WBP ancestors colonized the landscapes from multiple glacial refugia post glaciation [21,22, 23]. Our genetic data appear to support many of the inferences of post-glacial colonization originally drawn from patterns of mtDNA and cpDNA, which suggests that the northern Cascades in the US have been recently colonized from southern and western refugia [23]. The genotypes belonging to GG-1, GG-5, GG-7, GG-8, and GG-9 were mainly distributed in southern regions (SZ-4 to SZ-8) while the GG-4 genotypes were mainly distributed in the northern regions (SZ-1 to SZ-3, and BC) (Fig 1), suggesting that the region between SZ-3 and SZ-4 (around 46 degrees latitude) with extension to Columbia Gorge may be a major barrier to gene flow. This might also explain the drop in genetic diversity in northern WA and BC regions.

High genotypic diversity in three seed (sub) zones (SZ-2_E, SZ-4, and SZ-7_3) suggests that these regions may be candidate areas for selection of elite seed families with adaptation to pathogens/pests or other environmental stressors. However, admixtures have been detected in all the sampled seed zones, demonstrating that the trees examined in this study are heterogeneous. Genetically heterogeneous, admixed stands may have better fitness, providing candidate parent trees for breeding selection and restoration efforts.

Principal components of WBP genetic variations were recently detected with links to heterozygosity, latitude, and longitude in WBP stands [25]. In the present study principal component analysis revealed similar geographical trends in western North America (S2 Fig). Furthermore, assessment of individual trees for relative rust disease severity revealed that different genetic subgroups were associated with quantitative resistance to WPBR (Fig 8). Resistance screening programs identified several heritable traits as well as regional patterns for WBP resistance to *C*. *ribicola* [12]. These results indicate that the genetic components are important factors affecting WBP geographical distribution and resistance to pathogens/pests. A key goal of WBP breeding and conservation is to maintain high genetic diversity in the rust resistance programs to allow the species the best opportunity to evolve in the face of future abiotic and biotic challenges, including those of a changing climate. Rich genetic clines for adaptive traits provide a potential for precise genomic selection of WBP stands or seed families with predicted traits.

## Conclusion

This study reports on the development and application of WBP ns-SNP markers. Our SNP markers were developed by transcriptome comparison using RNA-seq technology in a core collection of seed families. These markers cover a wide range of expressed genes, and a large proportion of them produce amino acid changes in the putative proteins encoded by the genes, and thus a potential role in contributing to phenotypic variation in association studies. Using ns-SNP markers, genetic diversity of WBP seed families currently used in breeding and conservation programs were assessed. This study provides novel insights into the population structure of this endangered species. Experimental verification of a subset of ns-SNPs in high-throughput suggests that WBP genomic resources developed here may be invaluable in the future for functional genomics studies, population genetic study, germplasm resource assessment, and genome-wide association study in WBP and related five-needle pines.

## Supporting Information

S1 Table A list of seed families genotyped in the present study. (XLSX)

S2 Table Statistics of whitebark pine needle transcriptome de novo assembled from RNA-seq reads using the program Trinity. (XLSX)

S3 Table BLAST analysis of whitebark pine needle transcriptome. (XLSX)

S4 Table S4 Table: Flanking nucleotide sequences, SNP types, amino acid changes, and putative gene functions of 71 SNP loci genotyped by Sequenom iPLEX arrays. (XLSX)

S5 Table PCR primers and extension probes designed for 71 SNP loci genotyped by Sequenom iPLEX arrays. (XLSX)

S6 Table Summarized statistics for genetic variation of whitebark pine populations sampled in this study. (XLSX)

S7 Table F-statistics and estimates of Nm over all populations for each ns-SNP locus. (XLSX)

S1 Fig Functional classification of the coding DNA sequences (CDS) containing non-synonymous SNPs (ns-SNPs). CDS were derived from the whitebark pine transcriptome *de novo* assembled using RNA-seq reads. Gene annotation with GO terms was presented at the 2nd level for the biological processes.
(TIF)

S2 Fig A dendrogram generated by unweighted neighbor-joining method using genetic distance matrix based on SNP genotypic data, showing the relationship among all genotyped individual trees. Data in the phylogenetic dendrogram were drawn to scale with the branch length proportional to the genetic dissimilarity.
(TIF)

S3 Fig Association of principal components (PC-1 and PC-2) with geographic origins of the seed families. Genetic variations of 124 seed families were calculated by Principal Component Analysis based on genotypic data of 71 SNP loci. Above: PC-1 vs. latitude; Bottom: PC-2 vs. longitude.
(TIF)
