# Supplementary figures and images for: Genetic Diversity and Population Structure of Whitebark Pine (Pinus albicaulis Engelm.) in Western North America
Source: PLoS One. 2016 Dec 16;11(12):e0167986. doi: 10.1371/journal.pone.0167986 (PMC5161329; doi:10.1371/journal.pone.0167986)

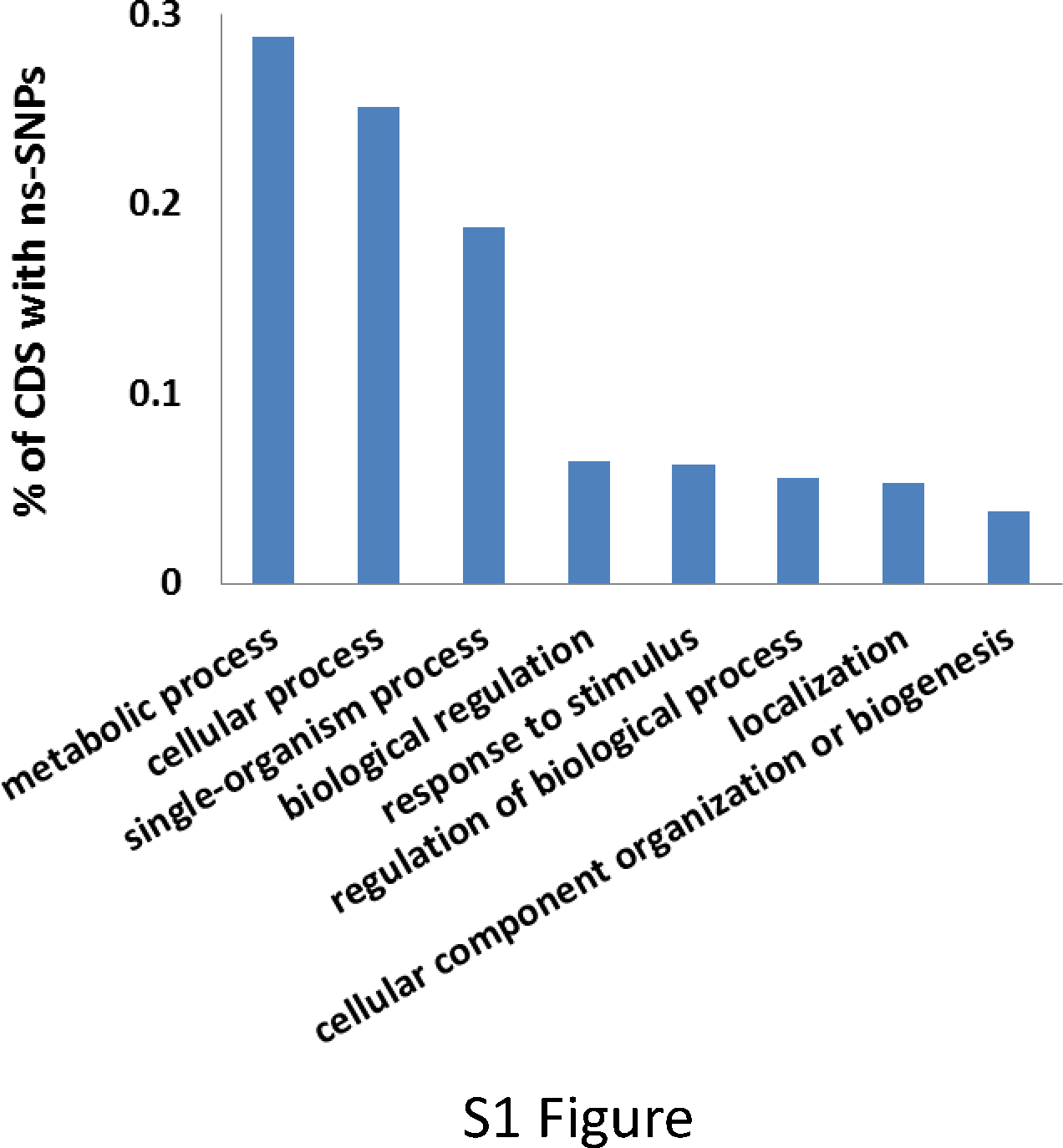

Supplement: S1 Fig — CDS were derived from the whitebark pine transcriptome de novo assembled using RNA-seq reads. Gene annotation with GO terms was presented at the 2nd level for the biological processes. (TIF) [file pone.0167986.s008.tif]

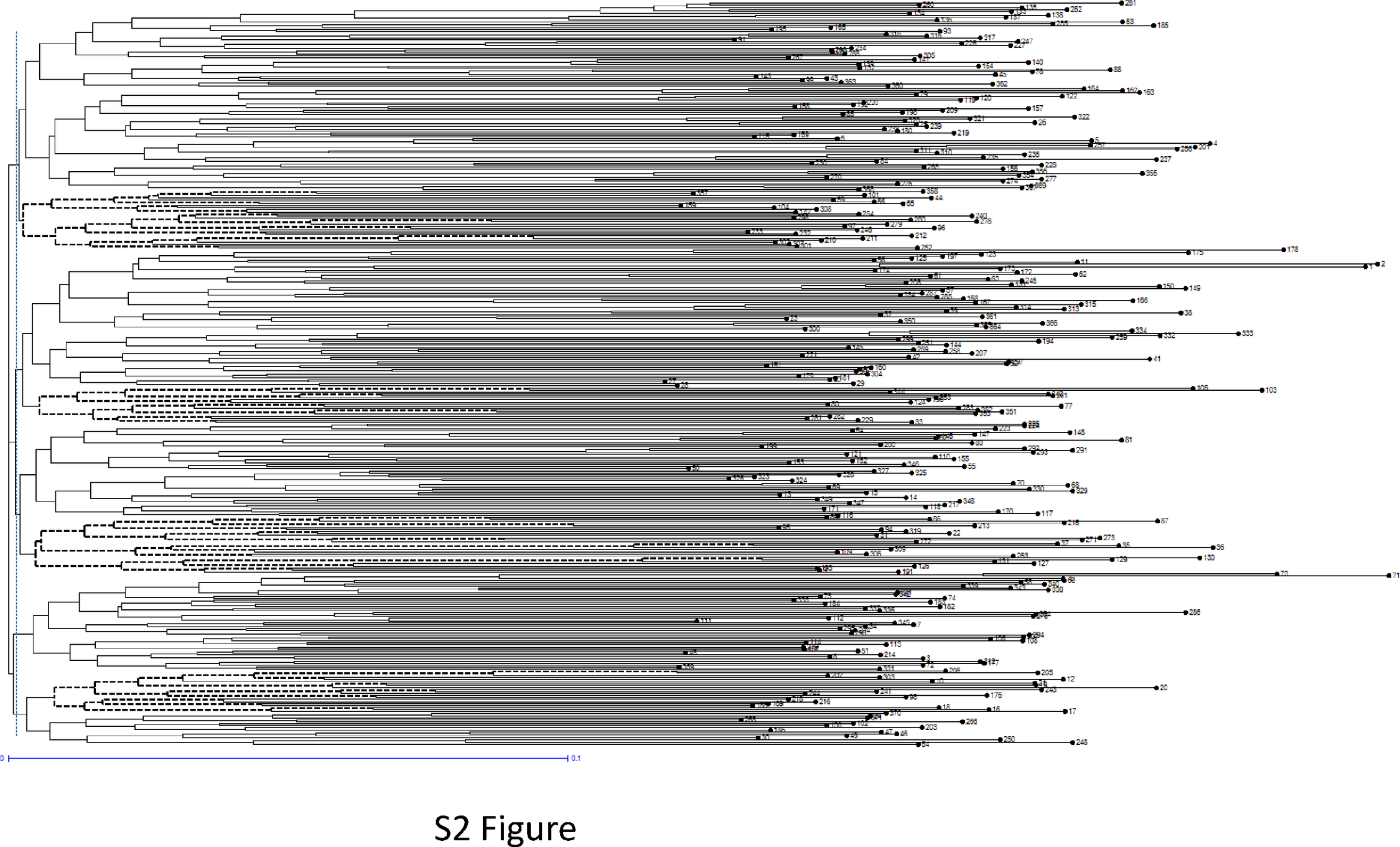

Supplement: S2 Fig — Data in the phylogenetic dendrogram were drawn to scale with the branch length proportional to the genetic dissimilarity. (TIF) [file pone.0167986.s009.tif]

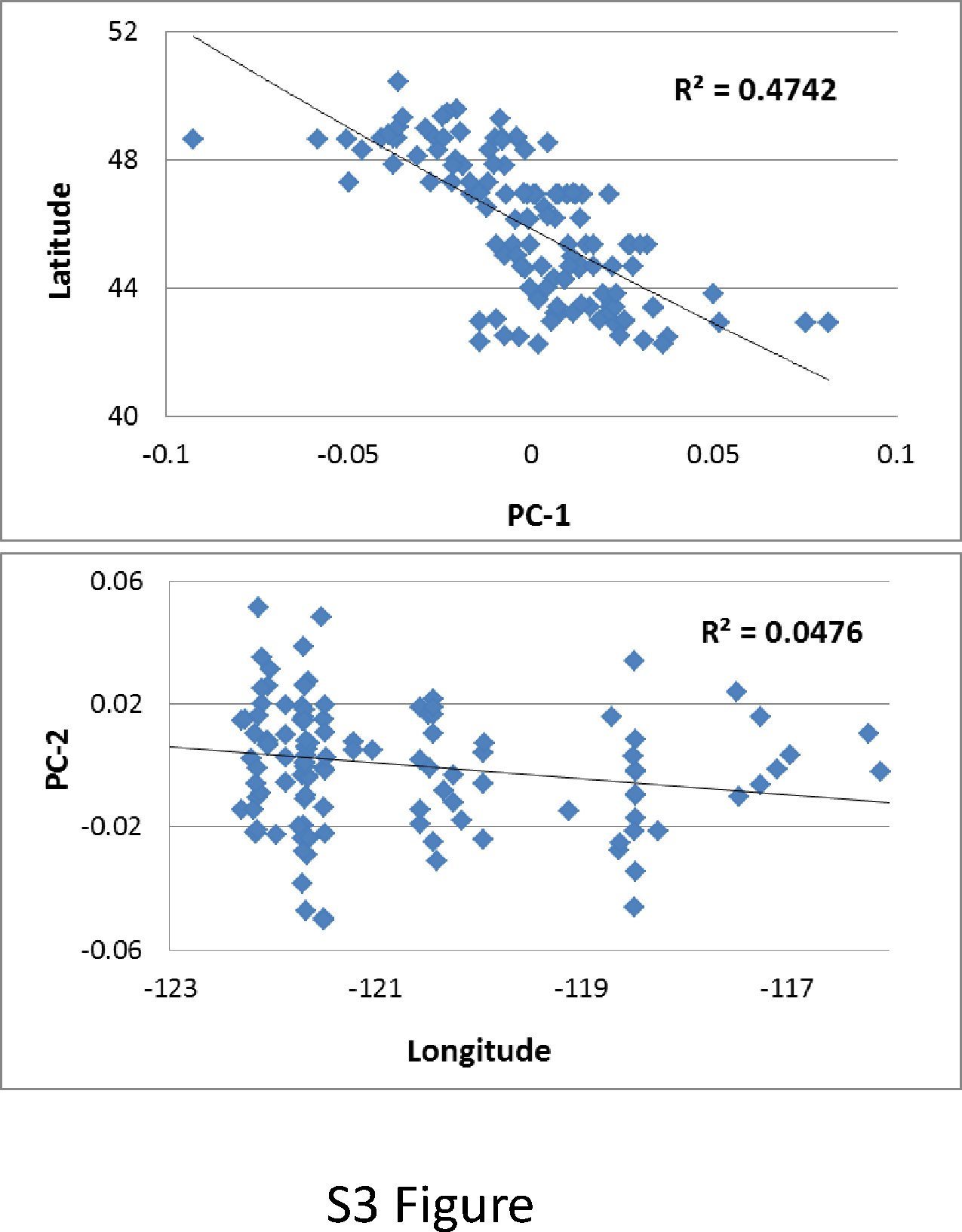

Supplement: S3 Fig — Genetic variations of 124 seed families were calculated by Principal Component Analysis based on genotypic data of 71 SNP loci. Above: PC-1 vs. latitude; Bottom: PC-2 vs. longitude. (TIF) [file pone.0167986.s010.tif]
